# Evolution of *Mycobacterium tuberculosis* Population Structure in Arkansas, 2009–2022, and Its Public Health Implications

**DOI:** 10.3390/microorganisms14102155

**Published:** 2026-09-24

**Authors:** Maria T. Marsh, Maheen Humayun, Zhenhua Yang

**Affiliations:** Department of Epidemiology, School of Public Health, University of Michigan, 1415 Washington Heights, Ann Arbor, MI 48105, USA

**Keywords:** tuberculosis surveillance, *Mycobacterial tuberculosis* genetic lineages, *M. tuberculosis* population structure evolution, tuberculosis elimination, low-incidence settings

## Abstract

The evolution of the *Mycobacterium tuberculosis* (*M.tb*) population structure in each population over time can help elucidate the drivers of tuberculosis (TB) epidemics. However, few previous studies have examined recent changes in *M.tb* population structure in low-incidence settings where TB elimination remains challenging. To fill this knowledge gap, we conducted a time-trend ecological study using deidentified TB surveillance and *M.tb* spoligotyping data on 696 incident TB cases diagnosed in Arkansas during 2009–2022. We calculated the yearly proportions of cases attributable to each of the major genetic lineages circulating in the state from 2009 to 2022 and assessed the association of *M.tb* genetic lineages with clinical characteristics using logistic regression models. The East Asian lineage contributed to 11.8% (7/60) of the cases in 2009, but this proportion increased to 30.2% (13/43) in 2022. Similarly, the proportion of the analyzed pulmonary TB (PTB) cases caused by the East Asian lineage increased from 14% (7/50) in 2009 to 30.5% (11/36) in 2022. The remarkable expansion of the East Asian lineage and the significant decline of the Euro-American lineage in Arkansas over time have important implications for TB elimination in the state.

## 1. Introduction

Despite its long history as a human disease, tuberculosis (TB) remains one of the world’s leading infectious killers [1]. To end the TB epidemic, a global plan to reduce TB incidence rate, the number of deaths caused by TB, and the financial burden associated with TB diagnosis, thereby eliminating TB as a public health threat by 2030, has been developed by the Stop TB Partnership. This partnership includes ~2000 international, non-governmental and governmental organizations, and patient groups [2]. Although significant progress in global TB elimination has been made since 2015, as shown by a 50% reduction of TB incidence in 2025, compared to the 2015 baseline, several challenges to advancing global TB elimination remain [1]. Eliminating TB in low-incidence states in the U.S. is a major challenge due to delayed diagnosis resulting from loss of expertise, redirection of public health resources away from TB programs towards other pressing public health issues, and unpreparedness for outbreaks in populations that are medically underserved or part of a minority group [3].

While TB affects every region of the world, *Mycobacterium tuberculosis* (*M.tb*), the causative agent of TB, has experienced geographical isolation, leading to *M.tb* population bottlenecks, resulting in the formation of seven distinct lineages. These include the Indo-Oceanic (Lineage 1), East Asian (Lineage 2), East-African-Indian (Lineage 3), Euro-American (Lineage 4), West African-1 (Lineage 5), West African-2 (Lineage 6), and *M. Bovis* lineages [4,5]. Currently, the most widespread lineages are the East Asian and the Euro-American lineages. The East Asian lineage tends to be more commonly found in East and Central Asia, Russia, and Southern Africa [6]. The Euro-American lineage is frequently found in Europe and the Americas, including the state of Arkansas, where the current study was conducted [6,7].

The outcomes of *M.tb* infection and disease vary, with some individuals being able to rapidly clear the infection, some containing the infection in a latent stage, and others quickly progressing to active disease that can present in various clinical forms [8]. Together with environmental and host-related factors, the genetic diversity of the *M.tb* pathogen plays a significant role in driving the differences in *M.tb* infection and disease outcomes [6,8]. Differences in disease severity, bacterial load, TB clinical manifestation, and transmission rate have been widely reported across the *M.tb* genetic lineages [9,10,11]. While the phenotypic impact of *M.tb* genetic diversity was evident from previous studies, epidemiological investigations have reported inconsistent lineage–phenotype associations across different populations [9].

Therefore, the aims of this study were to assess the recent changes in the *M.tb* population structure and to extend the knowledge base of the association of *M.tb* lineages with clinical characteristics. Given the differences in the epidemiological and phenotypic characteristics of the *M.tb* lineages, it is important to characterize the relative contribution of various *M.tb* lineages to the TB epidemic over time in each population. Characterizing the changes in *M.tb* population structure over time can help in developing mitigation strategies tailored to the changing dynamics of the remaining TB epidemic in different populations. A limited number of studies have used population-based data to describe the evolution of *M.tb* population structure changes over time and to characterize the clinical characteristics of *M.tb* lineages in low-incidence US states where TB elimination remains challenging [7,12,13]. Using 14 years of TB surveillance and *M.tb* genotyping data, we investigated the *M.tb* population structure changes over time in Arkansas, one of the low-incidence US states, and examined the association of *M.tb* lineages with important clinical characteristics of incident TB cases.

## 2. Materials and Methods

### 2.1. Study Population

This time-trend ecological study included a total of 696 *M.tb* isolates from 696 incident cases for which *M.tb* spoligotyping data was available. For patients who had more than one isolate genotyped, the isolate with the earliest specimen collection date was retained. These spoligotyped cases represent 91.2% (696/762) of all culture-confirmed incident TB cases and 64.1% (696/1084) of all incident TB cases reported to the Arkansas Department of Health (ADH) during 2009–2022.

### 2.2. Study Data

The study data included three types of variables: socio-demographic variables, clinical phenotype, and genetic lineage of *M.tb* isolates. Socio-demographic variables included age, gender, race/ethnicity, and nativity. Clinical phenotype was classified based on the anatomic site affected by TB. If TB affected only the lungs of a case, the case was classified as pulmonary TB (PTB). If TB affected a case’s organs other than their lungs, the case was classified as extrapulmonary TB (EPTB). If a case was diagnosed with both PTB and EPTB, the case was classified as concurrent PTB and EPTB (PTB-EPTB). All the cases with pulmonary TB, regardless of whether or not they had concurrent EPTB, were further classified into cavitary PTB or non-cavitary PTB cases, based on the findings of their chest X-ray radiography.

### 2.3. Data Analysis

We compared the characteristics of cases included and excluded in this study using chi-square tests. Among the 696 cases with genetic lineage information available, we calculated the yearly proportion of overall TB caused by each of the three most prevalent genetic lineages in Arkansas (Indo-Oceanic, East Asian, and Euro-American). We also calculated the proportion of PTB, EPTB, and PTB-EPTB attributable to each of the three lineages over time to assess the contribution of different *M.tb* genetic lineages to each TB clinical phenotype. To explore the role of nativity in driving the expansion of the East Asian lineage in Arkansas during the study period, we categorized TB cases with East Asian lineage into Asian foreign-born, non-Asian foreign-born, and US-born cases. We calculated the yearly numbers of Asian-born and other foreign-born cases among TB cases with East Asian lineage. Asian-born included individuals with place of birth indicated as Cambodia, China, Indonesia, Korea, Laos, the Philippines, Taiwan, Thailand, and Vietnam. To explore if certain TB clinical characteristics were more likely to be associated with certain *M.tb* genetic lineages, we evaluated two binary outcomes: TB anatomic site (PTB vs. EPTB) and cavitary PTB (yes/no). As described earlier, the EPTB category included PTB-EPTB to allow for comparison between different *M.tb* genetic lineages in their ability to cause disease outside of the lungs, the initial disease site of TB. Cavitary TB suggests advanced PTB with a greater likelihood of transmission [11]. Using logistic regression models, we determined crude and adjusted odds ratios (ORs) with 95% confidence interval (CI) for the two outcomes, respectively. The adjusted model included age, gender, race/ethnicity, and nativity. The East-African Indian and Bovis lineage were excluded from the analysis due to the small sample size. All statistical analyses were performed using SAS OnDemand for Academics (version 9.04).

## 3. Results

### 3.1. Characteristics of Study Population

The study population (n = 696) was racially diverse with 64.5% men and 66.8% U.S.-born persons (Table 1). The majority (81.2%) of the study cases had PTB alone, and 12.7% had only EPTB, while the remaining 6.0% had PTB-EPTB. The three most prevalent *M.tb* lineages that were found in this study population were Euro-American, East Asian, and Indo-Oceanic lineages.

Included and excluded cases were compared to assess any systematic differences between included versus excluded cases. We found that the distributions of age, race/ethnicity, nativity, and disease sites were different between included and excluded TB cases (*p*-value < 0.01) (Table 1). Those aged 0–14 were underrepresented in the study sample compared to those excluded (*p* < 0.0001). Non-Hispanic White and Non-Hispanic Black were overrepresented, while Native Hawaiian/Pacific Islander (NHPI) were underrepresented in the study sample compared to cases excluded (*p* < 0.0001). PTB cases were also overrepresented in comparison to those not included (*p* < 0.0001).

### 3.2. Time Trends of Overall and Subtypes of TB by M.tb Genetic Lineage

In 2009, the beginning of the study period, 80% (48/60) of the incident TB cases analyzed were caused by the Euro-American lineage, while the remaining 11.6% (7/60) and 6.7% (4/60) of the cases were caused by the East Asian and Indo-Oceanic lineages, respectively (Figure 1). The proportion of cases attributable to the Euro-American lineage reduced to 46.5% (20/43) by 2022, the end of the study period. Among PTB cases with genetic lineage information available, the East Asian lineage caused 14% (7/50) of PTB cases in 2009, and by 2022 it was the cause of 30.5% (11/36) of the PTB cases diagnosed in Arkansas (Figure 2).

Similarly, there was an increase in the proportion of EPTB cases caused by the East Asian and Indo-Oceanic lineages, especially from 2019 to the end of the study period. In 2022, 50% (1/2) of EPTB cases were caused by the East Asian lineage and 50% (1/2) were caused by the Indo-Oceanic lineage (Figure 3). The proportion of EPTB cases caused by the Euro-American lineage decreased from almost 100% (6/7) of cases in 2009 to 0% (0/2) of cases in 2022. The proportion of PTB-EPTB cases caused by the East Asian lineage increased over the study period (Figure 4). Specifically, it more than doubled from 11.4% in 2009 to 30.2% in 2022.

### 3.3. Relationship Between Nativity and Yearly Trend of East Asian Lineage-Associated TB

The trends of the yearly proportions of the East Asian lineage cases appeared to have certain correlations with the increase and decrease of the yearly numbers of the overall foreign-born cases among the East Asian lineage cases, although for some years, the parallel changes were not observed (Figure 5). It is also worth noting that the yearly number of the Asian East lineage cases who were foreign-born have increased during the study period.

### 3.4. Association of M.tb Genetic Lineage with Clinical Characteristics of TB

Cases attributable to the Bovis lineage were more likely to present with EPTB compared to the Euro-American lineage, and this association remained significant after adjusting for age, gender, race/ethnicity, and nativity (Table 2). U.S.-born persons were less likely to present with EPTB compared to non-U.S.-born persons.

The East Asian and Indo-Oceanic lineages were more likely to present with cavitary PTB compared to the Euro-American lineage; however, this association was not statistically significant either before or after adjusting for age, gender, race/ethnicity, and nativity (Table 3). Asian persons were less likely to present with cavitary PTB compared to Non-Hispanic White persons (aOR= 0.46, 95% CI: 0.21, 0.97).

## 4. Discussion

Previous studies showed that the distribution of genetic lineages of *M.tb* varies by geographic regions [14]. The evolution of the *M.tb* population structure in each population over time can help elucidate the drivers of TB epidemics. However, few population-based studies that examine the recent changes of *M.tb* population structure were conducted in low-incidence settings, like the state of Arkansas, where TB elimination remains challenging [7]. To fill this knowledge gap, we conducted a time-trend ecological study using deidentified TB surveillance and *M.tb* spoligotyping data on 696 incident TB cases diagnosed in Arkansas during 2009–2022. We calculated the yearly proportions of TB cases attributable to each of the major genetic lineages circulating in the state from 2009 to 2022 and assessed the association of *M.tb* genetic lineages with clinical characteristics using deidentified TB surveillance and *M.tb* spoligotyping data. Our study demonstrated a remarkable change in the *M.tb* population structure in Arkansas over time, characterized by a major decline in the proportion of incident TB cases attributable to the traditionally prevalent Euro-American Lineage as the traditionally less prevalent East-Asian Lineage expanded its presence.

Previous research has shown that *M.tb.* has co-evolved with humans for tens of thousands of years [15]. Studies using time-scale models demonstrate how modern human travel and geopolitical ties alter regional strain distribution over decades [14]. A previous study conducted in a high-TB-incidence district of Malawi involving isolates from 1687 patients diagnosed over a period of 15 years found a change in the proportion of cases caused by different lineages [16]. At the beginning of this study in 1995, the Euro-American lineage was the most common but by the end of 2010, the East-African-Indian lineage was more prevalent. However, unlike our study in which a remarkable expansion of the East Asian lineage was observed, the study in Malawi did not observe an increase in the number of the East Asian lineage cases in the study population. The observed differences between these two studies indicate the importance of regional studies for gaining insight into local population structure dynamics, given that the distribution of genetic lineages of *M.tb* varies across geographic regions and populations and evolves over time [16]. With TB affecting every region and all populations of the world, a better understanding of the evolving distribution of *M.tb* genetic lineages in each region or population is crucial for the development of new tools for effective TB prevention and control given that *M.tb* lineages differ in their ability to drive TB epidemics [9].

The East Asian lineage is the most widespread *M.tb* lineage globally and has two major clades, proto-Beijing and Beijing, with the Beijing clade originating in Beijing [17]. The Euro-American lineage has long been the predominant lineage in the region of the Americas [18]. An increase in the proportion of TB caused by the East Asian lineage in Arkansas may be a result of the increased migration from TB high-burden countries where the East Asian lineage is widespread, such as countries in East and Central Asia [5]. The small sample sizes of Asian-born cases in our study did not allow us to meaningfully assess the potential role of the Asian-born cases in the expansion of the East Asian lineage in our study, although a certain level of correlation between the trends of the yearly proportions of the East Asian lineage cases with the increase and decrease of the yearly numbers of the overall foreign-born cases among the East Asian lineage cases was observed for some of the study years (Figure 5).

It is also postulated that the successful global expansion of the East Asian lineage is a result of it being more virulent and pathogenic, compared to other lineages [19]. Even within the East Asian lineage, newer Beijing strains are more virulent than older proto-Beijing strains. This increase in virulence is due to the increased bacterial load in the lungs [20]. This notion of increased virulence is further supported by the increased fraction of PTB attributable to the East Asian lineage over time in this study as PTB primarily drives TB transmission from one human to another. Historically, the spread of the East Asian lineage has been associated with the spread of people, so there are many factors that could have contributed to this association [5]. Such an increase in TB caused by the East Asian lineage should be concerning for public health programs as East Asian lineage has been found to be associated with a higher risk of drug resistance, transmissibility, virulence, treatment failure, and relapse [9,19,21]. We also found that the East Asian lineage was more likely to present with cavitary PTB, although this association was not statistically significant. Cavitation in the lungs is a hallmark of TB pathogenesis and typically results in an increased risk of person-to-person transmission due to coughing and a high bacillary load [17]. Taken together, East Asian lineage is likely driving PTB over time and may be associated with TB transmission in Arkansas. Given TB transmission can also be driven by social factors, such as concentration of certain diasporas and living conditions, as well as biological factors, the increased prevalence of the East Asian lineage in many regions of the world, including Arkansas, may also be largely due to social factors.

Our study, which analyzed 91.2% (696/762) of all culture-confirmed incident TB cases reported for Arkansas during 2009-2022, was the first or one of the very few population-based studies evaluating *M.tb* population structure over time in a low-incidence setting where TB elimination remains challenging. In the present study the East Asian lineage contributed to 11.8% (7/60) of the cases in 2009, but this proportion increased to 30.2% (13/43) in 2022. Similarly, the proportion of the analyzed pulmonary TB (PTB) cases caused by the East Asian lineage increased from 14% (7/50) in 2009 to 30.5% (11/36) in 2022. The remarkable expansion of the East Asian lineage and significant decline of the Euro-American lineage in Arkansas over time has important implications for TB elimination in the state. Previous epidemiological studies conducted in Arkansas by Humayun et al. have highlighted the racial and ethnic disparities in the TB epidemic in Arkansas, suggesting that TB control in Arkansas can benefit from a targeted focus on groups that experience a greater risk of TB [22]. Additionally, molecular epidemiological investigation of the TB epidemic in Arkansas highlighted the role of ongoing community transmission among racial/ethnic minority groups [23]. The findings from the present study provide important additional insights into the role of genetic lineages in driving the TB epidemic. Taken together, these studies can help design a more holistic mitigation strategy that is informed by several different layers of evidence, including genetic lineage, health equity and transmission dynamics.

This study had several strengths. Unlike many previous studies that described *M.tb* population dynamics using hospital-based convenient samples, our study used statewide data spanning 14 years from 2009–2022 to identify the evolution of the local *M.tb* population structure in Arkansas. We used both patient surveillance and spoligotyping data to better understand the TB epidemic in Arkansas. This study not only generated important new knowledge about the evolution of TB epidemiology in the state of Arkansas with important implications for improving TB control but also holds important lessons for other low-incidence settings where TB elimination remains challenging. Populations where dynamics of TB epidemiology and the underlying *M.tb* population structure have evolved can particularly benefit from generating updated evidence for developing evidence-based TB control efforts. Population-based surveillance data can help reduce selection bias by capturing a representative study sample as compared to a single center or hospital-based study. In addition to exploring the temporal trends of *M.tb* lineages, this study investigated the association of *M.tb* lineages with key demographic and clinical variables after adjusting for potential confounders.

However, this study had a few limitations. About 389 incident TB cases were excluded from the analyses due to lack of genotyping information. The exclusion of these cases may have resulted in the loss of important genotyping information regarding the evolution of *M.tb* population structure. Those aged 0–14 were underrepresented in the study sample, compared to those excluded potentially due to the difficulty in obtaining diagnostic specimens from pediatric population [24]. Non-Hispanic (NH) White and NH Black were overrepresented while Native Hawaiian/Pacific Islander (NHPI) were underrepresented in the study sample, compared to excluded cases, potentially introducing selection bias. PTB cases were also overrepresented in comparison to those not included. In addition, the lack of data on patient immune status and underlying comorbidities in our study limited our ability to assess the potential effect of host factors on the association between genetic lineage and clinical characteristics. Disease trends may not always reflect true disease incidence as changes in diagnostic practices and surveillance efforts can influence disease detection. As this study used surveillance data, it does not capture patients with no access to healthcare. While this study may provide an analytical framework for other US states, the *M.tb* population structure in Arkansas may not be representative of other US states. In the absence of transmission related data, we could not study the contribution of East Asian lineage to ongoing TB transmission in Arkansas.

## 5. Conclusions

As the one of few population-based studies conducted to gain insight into *M.tb* clinical strain population structure changes over time in a low TB incidence state of the United States, the present study demonstrated that the *M.tb* population structure in Arkansas changed significantly over time. The findings of this study indicate that to progress towards the goal of TB elimination in Arkansas, its TB control program needs to pay special attention to the increased role of the East-Asian lineage in driving the remaining TB epidemic in the state and identify the drivers of the increased expansion of this lineage. The findings of this study also highlight the need for a better understanding of *M.tb* population structure evolution in other low-incidence settings where TB elimination remains challenging.

## Figures and Tables

**Figure 1:**
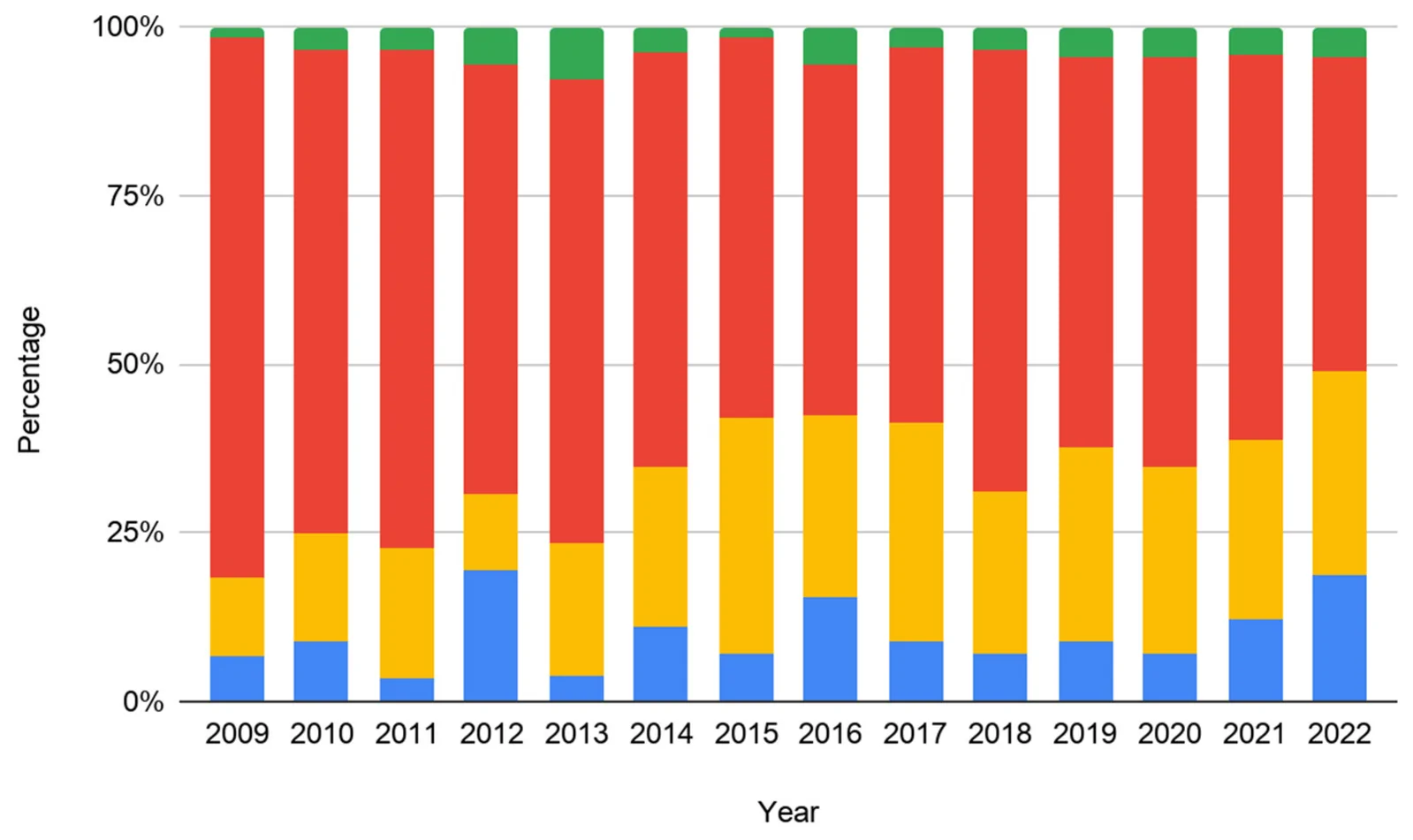
Time trends of the yearly proportions of the overall analyzed incident TB cases caused by major *Mycobacterium tuberculosis* (*M.tb*) lineages in Arkansas, during 2009–2022 (n = 696). Blue color: Indo-Oceanic lineage; yellow color: East Asian lineage; red color: Euro-American lineage; and green color: others.

**Figure 2:**
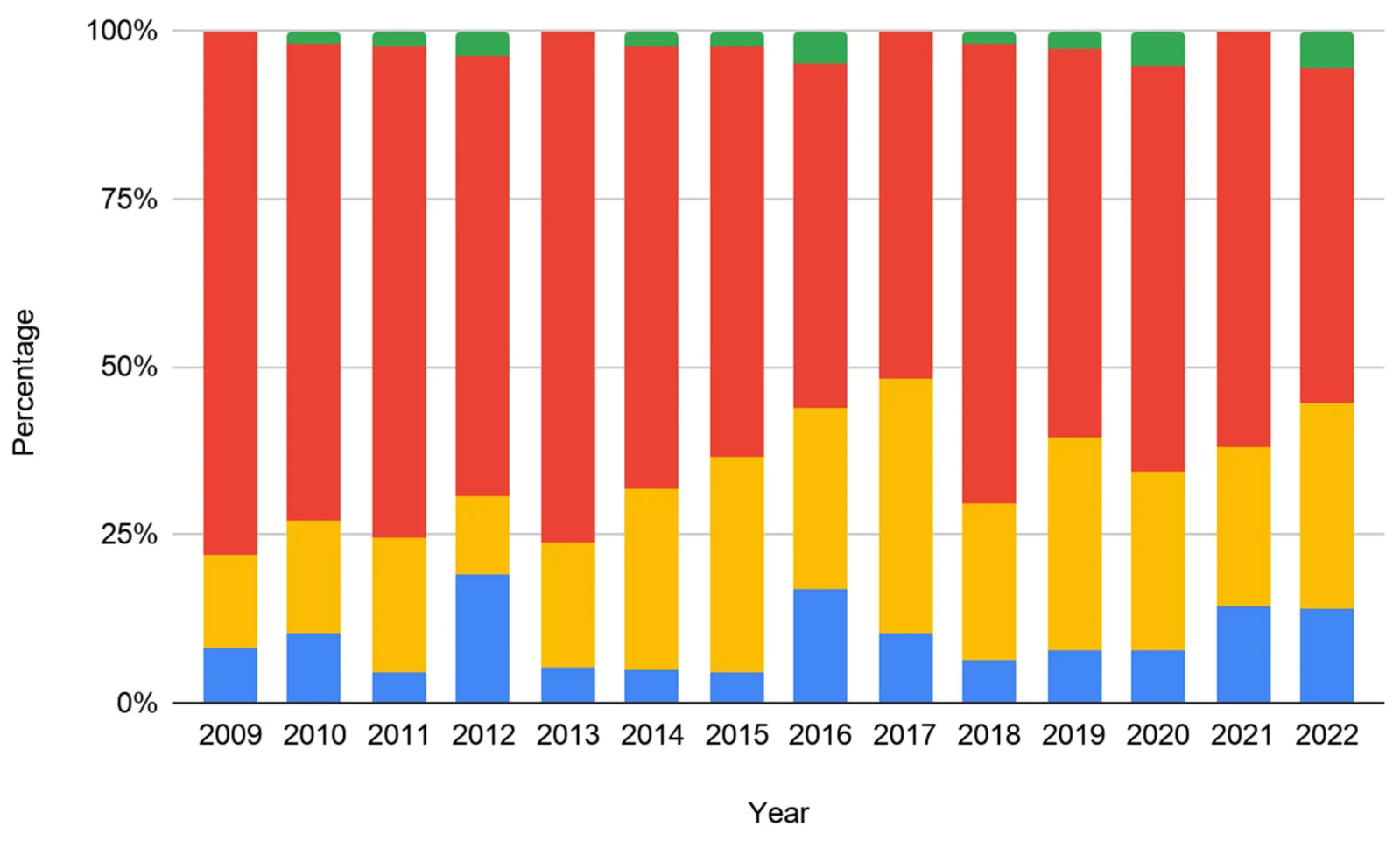
Time trends of the yearly proportions of the incident pulmonary TB cases caused by major *Mycobacterium tuberculosis* (*M.tb*) lineages in Arkansas, during 2009–2022 (n = 565). Blue color: Indo-Oceanic lineage; yellow color: East Asian lineage; red color: Euro-American lineage; and green color: others.

**Figure 3:**
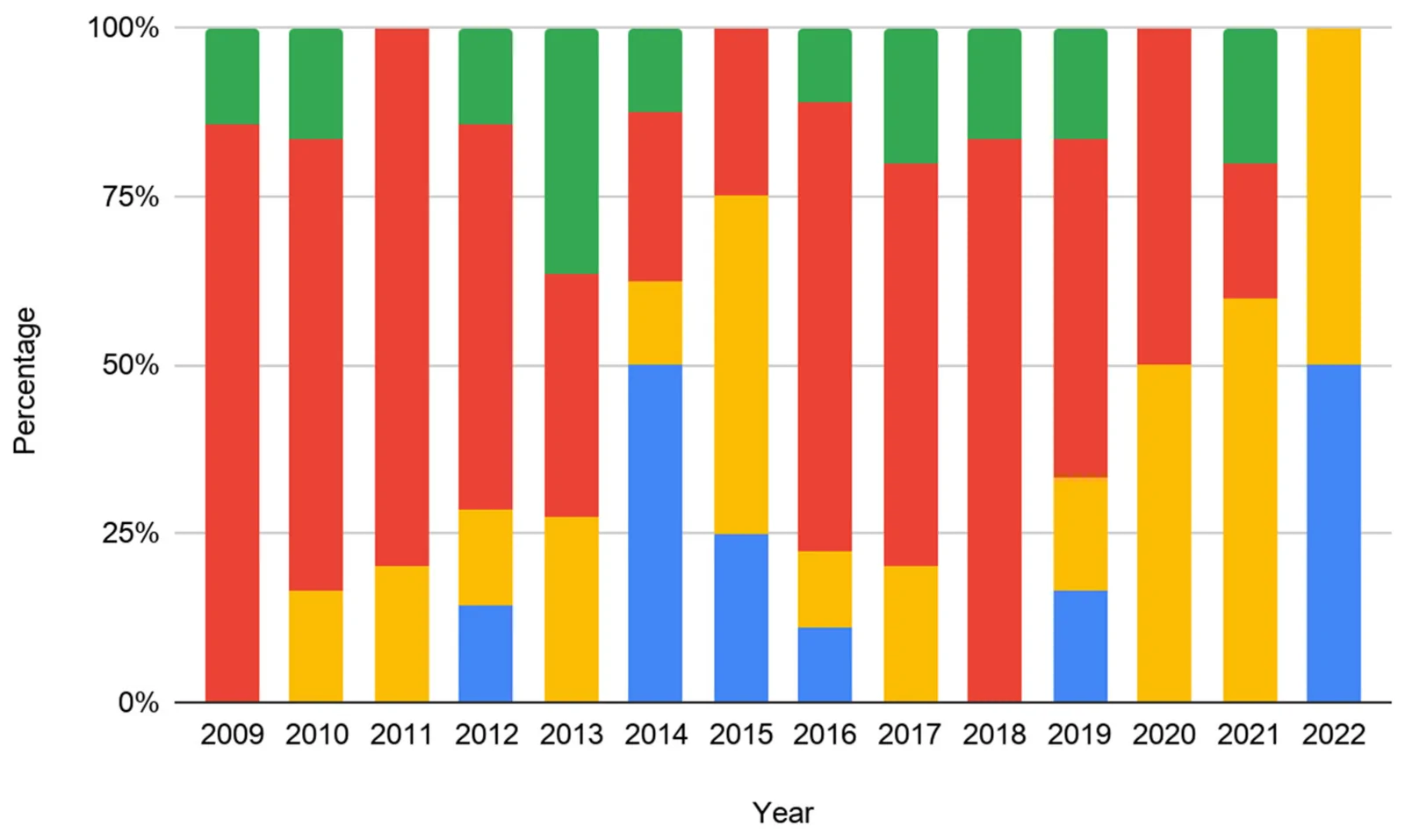
Time trends of the yearly proportions of the analyzed incident extrapulmonary TB cases caused by major *Mycobacterium tuberculosis* (*M.tb*) lineages in Arkansas, during 2009–2022 (n = 89). Blue color: Indo-Oceanic lineage; yellow color: East Asian lineage; red color: Euro-American lineage; and green color: others.

**Figure 4:**
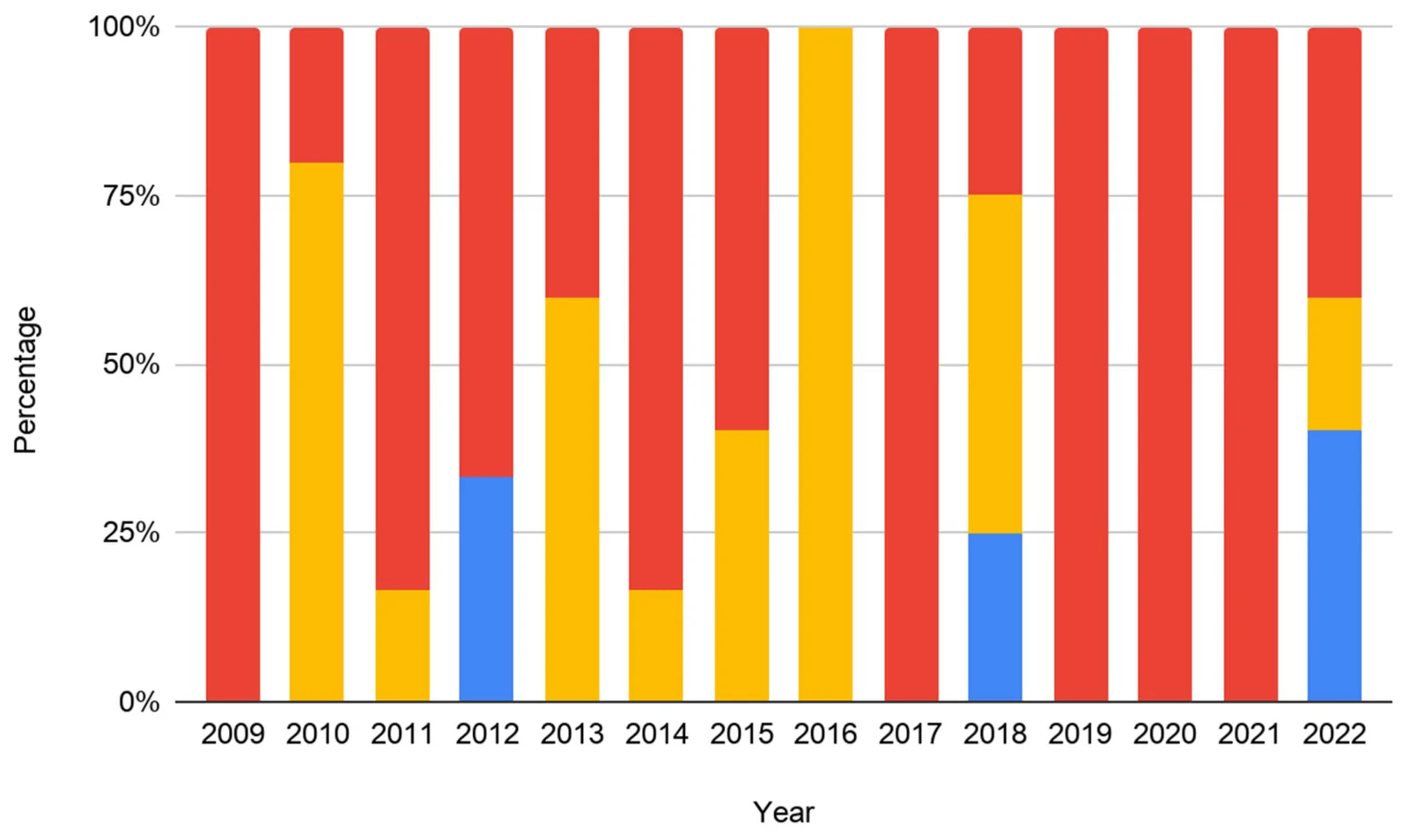
Time trends of proportions of the incident concurrent pulmonary and extrapulmonary TB cases caused by major *Mycobacterium tuberculosis* (*M.tb*) lineages in Arkansas, during 2009–2022 (n = 42). Blue color: Indo-Oceanic lineage; yellow color: East Asian lineage; red color: Euro-American lineage; and green color: others.

**Figure 5:**
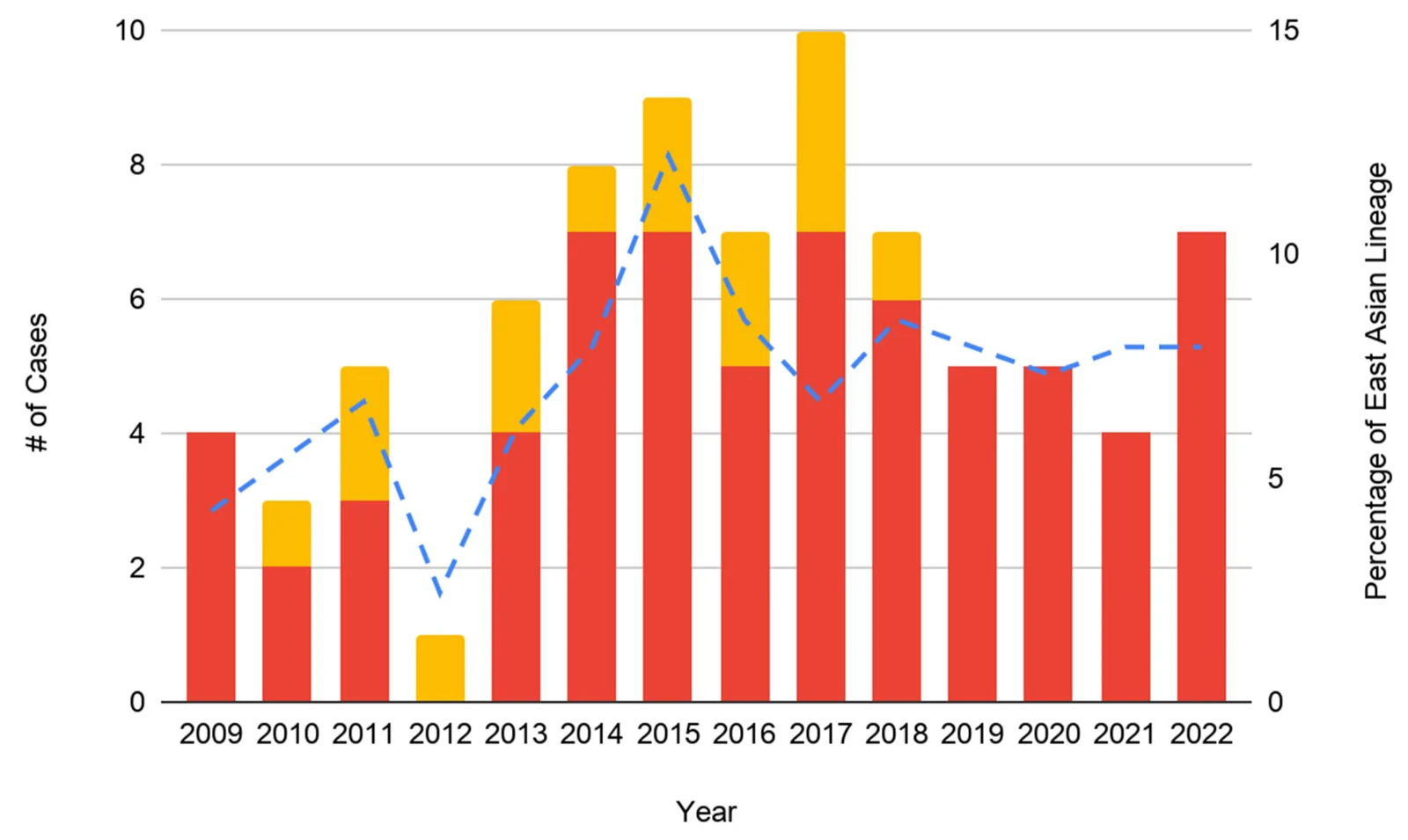
The yearly proportions of TB cases attributable to the East Asian lineage (blue dashed line, n = 164) and the yearly numbers of East Asian lineage cases who were (1) born in Asia (yellow bars, n = 15) and (2) born outside of the US other than Asia (red bars, n = 66).

**Table 1:** Socio-demographic, clinical, and microbial characteristics of the study sample comprising 696 tuberculosis (TB) incident cases diagnosed in Arkansas during 2009–2022 for which genetic lineage information was available and the 389 cases that were excluded from the study due to lack of genetic lineage information.

Variable	Included n (%)	Excluded n (%)	*p*-Value ^1^
Age (yrs)			<0.0001
0-14	14 (2.10)	93 (23.9)	
15–24	72 (10.4)	29 (7.5)	
25–64	415 (59.7)	193 (49.6)	
≥65	194 (27.9)	74 (19.0)	
Gender			0.2464
Female	240 (34.8)	149 (38.3)	
Male	449 (64.6)	239 (61.4)	
Unknown	6 (0.86)	1 (0.30)	
Race/Ethnicity			<0.0001
Hispanic	88 (12.7)	60 (15.4)	
Asian	72 (10.4)	51 (13.1)	
Non-Hispanic Black	200 (28.8)	86 (22.1)	
Native Hawaiian/Pacific Islander	79 (11.4)	82 (21.1)	
Non-Hispanic White	252 (36.3)	110 (28.3)	
U.S. Born			0.0236
Yes	464 (66.8)	233 (59.9)	
No	231 (33.2)	156 (40.1)	
Disease Site			<0.0001
Pulmonary	564 (81.2)	268 (68.9)	
Extrapulmonary	86 (12.4)	97 (24.9)	
Both	42 (6.0)	24 (6.17)	
Genetic Lineage			
Indo-Oceanic	66 (9.49)	n/a	
East Asian	164 (23.6)	n/a	
Euro-American	437 (62.9)	n/a	
Other ^2^	28 (4.03)	n/a	

^1^ Chi-Squared. ^2^ ‘Other’ consisted of *M. Bovis*, *M. Bovis*-BCG, East-African-Indian (L3) and mixed.

**Table 2:** The associations between *Mycobacterium tuberculosis* (*M.tb*) genetic lineage and TB disease site of TB (EPTB ^1^ vs. PTB) assessed by analyzing 696 incident cases diagnosed in Arkansas during 2009–2022 using logistic regression models.

Variable	Unadjusted OR (95% CI)	Adjusted OR (95% CI)
Age (yrs)		
0–14	1.66 (0.45, 6.07)	2.11 (0.55, 8.20)
15–24	ref	ref
25–64	0.88 (0.47, 1.67)	0.98 (0.51, 1.87)
≥65	1.08 (0.55, 2.12)	1.15 (0.56, 2.40)
Gender		
Female	ref	ref
Male	0.99 (0.73, 1.35)	0.92 (0.61, 1.38)
Race/Ethnicity		
Hispanic	0.84 (0.43, 1.65)	0.44 (0.14, 1.41)
Asian	1.50 (0.80, 2.84)	1.11 (0.31, 4.05)
Non-Hispanic Black	1.10 (0.68, 1.79)	1.21 (0.71, 2.05)
Native Hawaiian/Pacific Islander	1.76 (0.97, 3.20)	1.16 (0.34, 4.00)
Non-Hispanic White	ref	ref
U.S. Born		
Yes	1.16 (0.34, 4.00)	0.44 (0.26, 0.77)
No	ref	ref
Genetic Lineage		
Indo-Oceanic	1.30 (0.68, 2.47)	0.95 (0.47, 1.93)
East Asian	1.04 (0.65, 1.66)	0.73 (0.42, 1.27)
East-African-Indian	2.07 (0.52, 8.18)	1.31 (0.32, 5.42)
Euro-American	ref	ref
*M. bovis*	5.63 (1.84, 17.2)	7.42 (2.25, 24.4)

^1^ Cases with EPTB and PTB-EPTB were classified as EPTB cases.

**Table 3:** Association between *Mycobacterium tuberculosis* (*M.tb*) genetic lineage and cavitary pulmonary TB (cavitary pulmonary TB vs. non-cavitary pulmonary TB) using logistic regression models (n = 607), Arkansas, 2009–2022.

Variable	Unadjusted OR (95% CI)	Adjusted OR (95% CI)
Age (yrs)		
0–14	0.17 (0.02, 1.44)	0.19 (0.02, 1.63)
15–24	ref	ref
25–64	1.43 (0.82, 2.48)	1.47 (0.85, 2.57)
≥65	0.72 (0.39, 1.33)	0.75 (0.40, 1.43)
Gender		
Female	ref	ref
Male	1.05 (0.74, 1.48)	1.07 (0.76, 1.51)
Unknown	0.32 (0.04, 2.75)	0.34 (0.04, 2.95)
Race/Ethnicity		
Hispanic	1.49 (0.89, 2.50)	1.35 (0.79, 2.32)
Asian	0.70 (0.37, 1.31)	0.46 (0.21, 0.97)
Non-Hispanic Black	1.22 (0.81, 1.83)	1.10 (0.72, 1.70)
Native Hawaiian/Pacific Islander	1.39 (0.78, 2.44)	0.99 (0.51, 1.94)
Non-Hispanic White	ref	ref
U.S. Born		
Yes	1.59 (1.02, 2.51)	1.59 (1.02, 2.52)
No	ref	ref
Genetic Lineage		
Indo-Oceanic	1.25 (0.71, 2.20)	1.14 (0.63, 2.04)
East Asian	1.25 (0.85, 1.85)	1.20 (0.79, 1.80)
East-African-Indian	0.66 (0.13, 3.47)	0.63 (0.12, 3.30)
Euro-American	ref	ref
*M. bovis*	0.83 (0.15, 4.59)	0.99 (0.16, 6.07)

## Data Availability

The data presented in this study are available on request from the corresponding author. The data are not publicly available as data can be shared with other research teams after the review and approval of their research proposal by the Arkansas Department of Health’s Scientific Advisory Committee.

## Abbreviations

The following abbreviations are used in this manuscript.

*M.tb*	*Mycobacterium tuberculosis*
CDC	Centers for Disease Control
NHPI	Native Hawaiian/Pacific Islander
ADH	Arkansas Department of Health
PTB	Pulmonary TB
EPTB	Extrapulmonary TB
PTB-EPTB	Concurrent PTB and EPTB
MIRU-VNTR	Mycobacterium Interspersed Repetitive Unit-Variable Number Tandem Repeats
LTBI	Latent TB infection
